# Genesis and Maintenance of Attentional Biases: The Role of the Locus Coeruleus-Noradrenaline System

**DOI:** 10.1155/2017/6817349

**Published:** 2017-07-20

**Authors:** Mana R. Ehlers, Rebecca M. Todd

**Affiliations:** ^1^Department of Psychology, University of British Columbia, 2136 West Mall, Vancouver, BC, Canada V6T 1Z4; ^2^Djavad Mowafaghian Centre for Brain Health, University of British Columbia, 2215 Wesbrook Mall, Vancouver, BC, Canada V6T 1Z3

## Abstract

Emotionally arousing events are typically better remembered than mundane ones, in part because emotionally relevant aspects of our environment are prioritized in attention. Such biased attentional tuning is itself the result of associative processes through which we learn affective and motivational relevance of cues. We propose that the locus coeruleus-noradrenaline (LC-NA) system plays an important role in the genesis of attentional biases through associative learning processes as well as their maintenance. We further propose that individual differences in and disruptions of the LC-NA system underlie the development of maladaptive biases linked to psychopathology. We provide support for the proposed role of the LC-NA system by first reviewing work on attentional biases in development and its link to psychopathology in relation to alterations and individual differences in NA availability. We focus on pharmacological manipulations to demonstrate the effect of a disrupted system as well as the *ADRA2b* polymorphism as a tool to investigate naturally occurring differences in NA availability. We next review associative learning processes that—modulated by the LC-NA system—result in such implicit attentional biases. Further, we demonstrate how NA may influence aversive and appetitive conditioning linked to anxiety disorders as well as addiction and depression.

## 1. Introduction

Emotional salience enhances both attention and memory. For example, we typically remember emotionally arousing events better than mundane ones, reliving the birth of a child or a teenage humiliation with a high degree of vividness decades later [1–3]. We remember these events better in part because we pay heightened attention to emotionally relevant aspects of our environment that signal potential punishment and reward [4, 5]. In turn, such patterns of heightened attention are themselves the result of emotional learning processes that tune our perceptual systems to prioritize such affectively and motivationally relevant cues (e.g., [6–8]). Visual selective attention, or *attentional prioritization*, is the process by which we tune ourselves to the world so that, of the millions of bits per second transmitted by the retina [9], the information that is most important, or salient to us, reaches awareness and guides action. *Affect-biased attentional prioritization* [10], or selective prioritization of what is emotionally or motivationally relevant, can be highly adaptive, as emotional arousal signals events that are important to attend and remember in the interest of survival. Yet at the extreme ends of the spectrum, affect-biased attentional prioritization of specific categories of stimulus, which are often unconscious and automatic, is symptomatic of psychopathology. For example, implicit biases toward stimuli associated with threat characterize anxiety disorders [11], and biases to attend trauma-related cues characterize posttraumatic stress disorder (PTSD) [12]. According to popular models of PTSD, such trauma-related biases are themselves the result of Pavlovian associative learning processes [13]. Moreover, altered biases in attention to reward-related cues are linked to both depression [14, 15] and addictive behaviours [16–18]. In addiction as well, biases to addictive cues are thought to result from learning associations between the cue predicting reward and the actual reward [16]. It should be noted that affectively biased attentional prioritization is only one of several forms of *attentional bias* studied in relation to psychopathology. Indeed, whereas attentional prioritization measures preexisting filters that inform what we will see of the world before we ever encounter it, many clinical studies have focused on another form of attentional bias: difficulty with *attentional disengagement* from salient stimuli once they have already been observed [19]. In this paper, we will focus on the role of the locus coeruleus (LC) and noradrenaline (NA) system in the less-explored domain of attentional prioritization, as well as the ways in which emotional learning processes can give rise to specific habits of attentional tuning. Evidence directly linking the LC-NA system to maladaptive patterns of emotional learning associated with attentional biases in psychopathology is sparser. With that caveat, we will review convergent evidence for hypotheses about the role of NA in posttraumatic stress disorder, depression, and addiction and highlight future research directions to establish more direct links.

## 2. Attentional Biases

### 2.1. Attentional Biases in Development

Attentional biases appear early in development and specific biases predict later emergence of a range of maladaptive outcomes. A body of recent research has focused on the etiology of maladaptive attentional biases in childhood and adolescence and has suggested a causal role for such biases in the development of anxiety disorders [20]. Research by Perez-Edgar and colleagues has examined the role of attentional bias in moderating the link between temperament and psychopathology over development. Their research points to attentional biases observed early in development as a key mechanism linking temperamental inhibition—a temperament style associated with shyness, which involves higher levels of fearful responses to novel environmental stimulation measurable at birth—to later social withdrawal and anxiety. For example, behavioural inhibition in toddlers has predicted later social withdrawal in children who showed an attentional bias to threat at 5 years old [21], and attention bias to threat in adolescence has predicted adolescent social withdrawal [22]. Such developmental patterns also extend to biases towards reward. Temperamental exuberance is linked to both externalizing problems and attentional bias to reward in children [23]. Convergent evidence suggests a link between attention bias in development and vulnerability to substance abuse. In adolescence, externalizing problems are strongly associated with substance abuse problems [24], and in adulthood, a history of addiction has been linked to generalized enhancement of attentional bias for reward [17]. To date, development of individual differences in attentional bias associated with anxiety and depression has been primarily linked to individual differences in serotonergic function and variation in the 5HTTLPR region of the serotonin transporter gene—albeit only in some populations and in certain contexts [25, 26]. Yet, not only have findings been equivocal, but most of these studies have focused on biases operationalized as difficulties in disengaging attention [19]. We propose that NA plays a crucial role in implicit attentional prioritization, rather than effortful disengagement of attention. Specifically, we suggest that it may play a role in both the genesis and maintenance of such selective attentional biases as they are tuned by life experience.

### 2.2. The Role of NA in Biasing Attentional Prioritization

**Figure 1:**
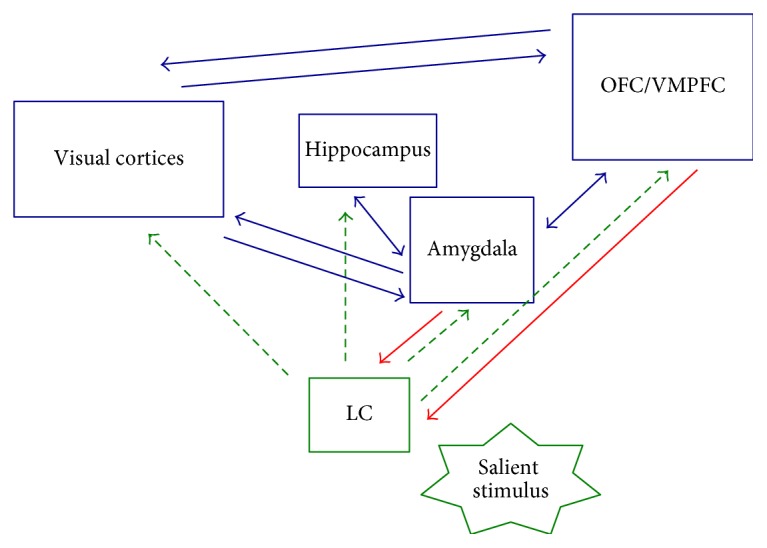
Key pathways emphasized by the biased attention by norepinephrine (BANE) model: green dashed lines indicate noradrenaline (NA) pathways. Red lines indicate projections to the locus coeruleus (LC). Thicker lines indicate direct modulation of visual cortex activity in affect-biased attention. NA activity is implicated in both stimulus-encoding and selective attention [27]. A salient stimulus activates locus coeruleus (LC) neurons, which project widely to cortical and subcortical regions. Adapted with permission from “Neural and genetic processes underlying affective enhancement of visual perception and memory” by Markovic et al. [4]. Copyright 2014 by Elsevier.

**Figure 2:**
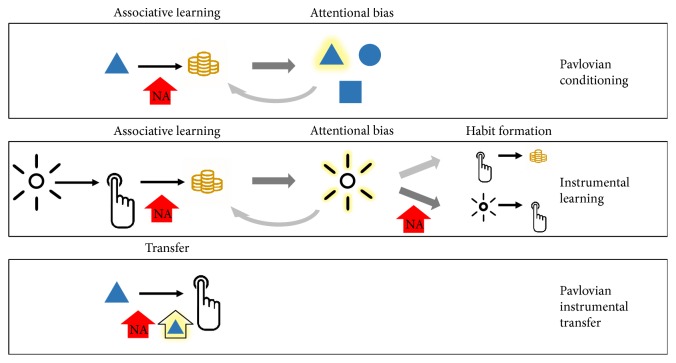
Overview over how (appetitive) noradrenaline (NA) modulated associative learning may give rise to attentional biases. In a simple Pavlovian conditioning paradigm, NA may influence associative learning processes leading to attentional bias for conditioned stimuli (CS) (for both appetitive and aversive unconditioned stimuli). In instrumental learning, initial learning of action-outcome relation is affected by NA giving rise to attentional biases for action-triggering stimuli. In a second step, attentional biases and noradrenergic processes may bias behaviour towards the habitual by strengthening stimulus-response over action-outcome relations. Finally, Pavlovian CS can influence instrumental responding, a process that may be influenced by both NA and attentional biases to the CS. Enhanced associative learning can manifest in excessive attentional biases characteristic of psychopathology.

Although the role of NA in guidance of attention to salient aspects of the environment has been thoroughly reviewed elsewhere ([4, 27–29]), we recapitulate some key points here. The LC-NA system has been found to play a key role in modulation of visual attention to salient aspects of the world (for review, see [4]) (Figure 1). NA neurons in the LC are phasically activated by salient environmental events, including visually salient, novel, task-relevant, or emotionally salient stimuli [30–32], resulting in release of NA. Such phasic LC activity has been associated with selective attention to relevant stimuli [33]. Current theoretical models suggest that phasic NA specifically plays a role in modulating neural gain associated with biased competition processes, reducing the threshold of sensory neurons to cues that are *relevant* either due to explicit task-related demands, visual salience, or motivational/affective salience acquired through life experience [4, 29, 34], while raising the threshold for neurons processing irrelevant ones. Phasic NA activity is thus thought to increase discrimination between relevant and irrelevant environmental information [35], improving the signal-to-noise ratio for relevant stimuli [36]. In their recent GANE model, Mather and colleagues have further emphasized interactions between glutamate and NA processes in creating hotspots that modulate effects of arousal on learning and memory [29]. Yet, LC-NA activity is also important for sensor-gating processes by which silent neurons become responsive to relevant stimuli, with additional neurons recruited in a process that does not necessarily require suppression of surrounding neurons [27, 37]. Importantly, LC-NA activity plays a role in establishing biases for particular categories of stimulus via associative learning (Figure 2). LC neurons can initially fire in response to direct reward and punishment and subsequently fire to any stimuli associated with the salient event [27]. Studies in rodents suggest that, in development, when noradrenergic alpha2b receptors mature, emotional learning is strongly reduced [38]. Moreover, modulation of long-term changes in synaptic strength and gene transcription allows the NA system to guide behaviour based on stimulus salience within a given context [39].

**Figure 3:**
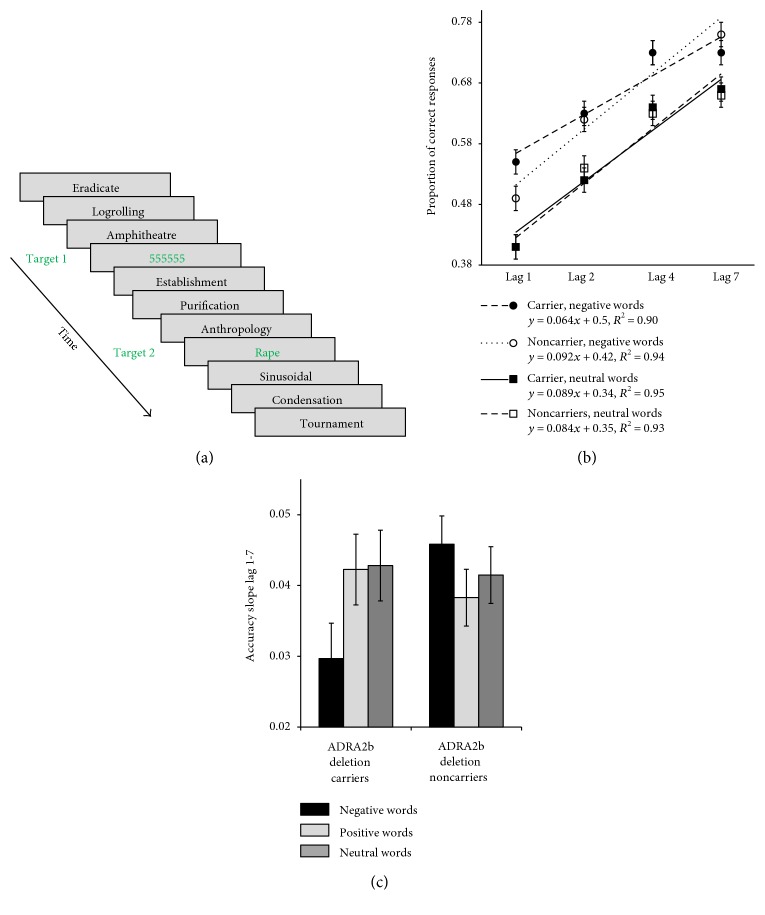
(a) Sample trial in attentional-blink (AB) task. Two targets were presented among several distractors: target 2 was a positive, negative, or neutral word. It was presented after target 1 after zero (Lag 1), one (Lag 2), three (Lag 4), or six (Lag 7) distractors. At the end of each trial, participants had to report both targets. (b) Proportion of correct responses for *ADRA2b* deletion carriers and noncarriers as a function of the lag between the two targets and emotion category. (c) The slope for accuracy from Lag 1 to Lag 7 as a function of group and emotion category. Adapted from “Genes for emotion-enhanced remembering are linked to enhanced perceiving” by Todd et al. [41]. Copyright 2013 by Sage Publications.

**Figure 4:**
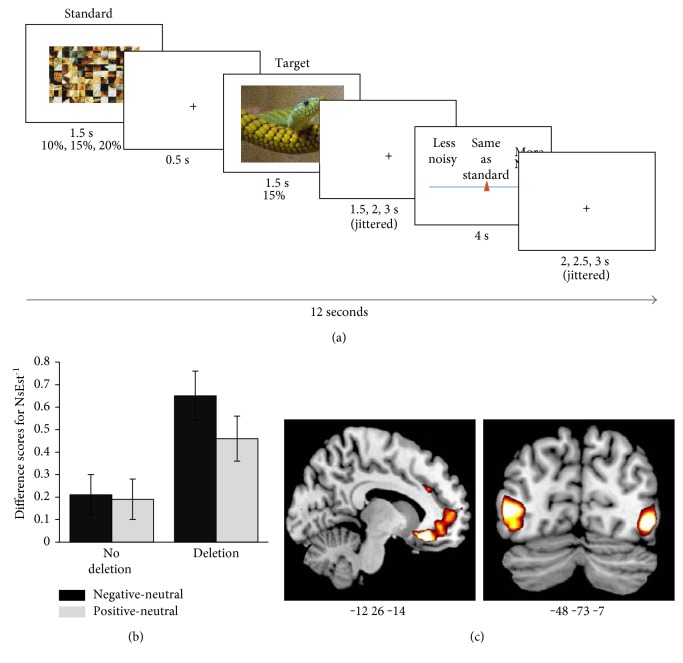
(a) Noise Estimation task to determine emotionally enhanced vividness (EEV). A standard (scrambled image) was overlaid with varying levels of noise. The standard was followed by the target overlaid with 15% noise. Participants were asked to indicate whether the target had more or less noise relative to the standard. (b) Difference scores for ratings of inverse noise estimation (NsEst^−1^), a measure of perceptual vividness for negative and positive > neutral stimuli in noncarriers and carriers of the *ADRA2b* deletion variant. Deletion carriers show greater EEV than noncarriers. (c) Statistical maps showing parametric modulation by EEV in the ventromedial prefrontal cortex for *ADRA2b* carriers > noncarriers and in the lateral occipital complex showing modulation by EEV across both groups. Adapted with permission from “Neurogenetic variations in norepinephrine availability enhance perceptual vividness” by Todd et al. [40]. Copyright 2015 by the Society for Neuroscience.

Our own research has contributed to a body of evidence indicating that biologically conferred differences between individuals, including genetic variations influencing NA activity, are associated with affect-biased attention to either emotionally arousing stimuli in general or positively or negatively valenced stimuli in particular [40–44]. In humans, genotyping for a common (~50%) deletion variant of the *ADRA2b* gene, which codes for alpha2b NA receptors and is thought to be associated with higher levels of intercellular NA [45, 46], provides a tractable window into the role of naturally occurring differences in NA availability on human cognitive endophenotypes. Building on previous research establishing a role for *ADRA2b* in emotional enhancement of memory, we used genotyping to examine the role of NA in affectively biased attentional prioritization, which might partly account for emotional enhancement of memory effects. As enhanced encoding of emotionally salient stimuli has been found to predict both subsequent recall and recognition memory (e.g., [47]), we hypothesized that carrying the deletion variant would be associated with a priori attentional tuning to emotional stimuli, resulting in higher likelihood of encoding emotionally salient stimuli. One method of measuring attentional prioritization is with an attentional blink paradigm (Figure 3). In this experiment, in every trial, an observer is faced with a rapid stream of stimuli and from it has to report two targets. When the second target (T2) appears within 500 ms of the first, observers are typically unable to report it [48]. This is called the *attentional blink*, because it is as if the mind blinks while neurocognitive resources are still tied up in encoding the first target (T1). Yet, when T2 is emotionally salient, the attentional blink is somewhat reduced, in a robust finding we refer to as *emotional sparing*. Following on the work of Di Lollo and colleagues [49], we have proposed that emotional sparing reflects implicit attentional tuning that facilitates awareness of emotionally relevant stimuli. Crucially, we have found that, whereas both carriers and noncarriers of the *ADRA2b* deletion variant show emotional sparing for both positive and negative stimuli, deletion carriers show an even greater sparing effect for negative stimuli, indicating a role for naturally occurring NA differences in biases in attentional prioritization [41] (Figure 3). Thus, putatively higher levels of NA availability were associated with attentional prioritization of affectively salient stimuli, such that they were more likely to be perceived, relative to neutral stimuli, in the first place. In an additional study, we showed participants positively and negatively arousing as well as low arousal scenes and measured recognition memory for the images in a surprise memory task one week later. Here, we found that enhanced subjective ratings of stimulus arousal during encoding were linked to enhanced memory one week later in deletion carriers only. Thus, putative differences in NA availability were associated with a stronger pattern of emotional enhancement of memory. These findings were consistent with nonhuman animal findings indicating that higher NA availability at encoding interacts with NA-mediated consolidation processes to produce enhanced memory for emotional events (for review, see [50]). Our own biased attention by norepinephrine (BANE) model emphasizes the role of the LC-NA system in brain circuits that mediate guidance of visual attention to emotionally salient stimuli, focusing on modulation of visual cortex by brain systems centered on the amygdala, ventromedial prefrontal cortex (VMPFC), and LC [4] (Figure 1). In a functional magnetic resonance imaging (fMRI) study, we found that *ADRA2b* deletion carriers subjectively perceive emotionally salient stimuli to be more perceptually vivid (higher signal-to-noise ratio) relative to neutral stimuli than noncarriers [40] (Figure 4). This effect of emotionally enhanced vividness (EEV) is associated with amygdala modulation of the visual cortex [47]. Consistent with the nodes of brain networks emphasized by the BANE model, this effect of putatively greater NA availability on EEV was associated with enhanced activity in hubs of the BANE network, particularly VMPFC (Figure 3). The prevalence of the *ADRA2b* deletion variant makes it a tractable tool for examining naturally occurring NA variation-related activity of alpha2b receptors in humans. However, other receptor subtypes also play an important role in modulating NA's effects on cognition. A substantial amount of animal research has demonstrated the importance of high affinity alpha2 and lower affinity alpha1 receptors for optimal functioning of the prefrontal cortex (PFC). More specifically, it has been shown that moderate levels of NA promote PFC functions such as working memory and top-down attention mechanisms as well as decision-making and emotion regulation (for review, see [51, 52]). Thus, it is likely noradrenergic activity at that these receptors also play a role in biased attention and learning.

### 2.3. Attentional Bias as Product of Emotional Learning

As mentioned above, implicit biases in attentional prioritization not only influence what we encode and remember but they are also themselves the product of learning and memory (Figure 2). Our research has found that in “real life,” the categories of stimulus for which attentional selection is biased are strongly shaped by traumatic experiences. Through these experiences, neutral stimuli are linked to high emotional arousal through associative learning processes [12, 53]. Moreover, the degree of this bias predicts PTSD diagnosis and is highly correlated with anxiety symptoms. Such examples of high-arousal associative learning experiences mirror effects found in controlled laboratory experiments using fear conditioning and complement a wide literature linking fear conditioning to anxiety disorders [54–57]. On the other end of the valence spectrum, attentional biases for substance-related stimuli, or cues, which predict craving in addiction can also be created through classical conditioning processes [58, 59]. Thus, considerable evidence suggests that attentional biases towards specific categories of salient stimuli develop through associative learning processes, and they do so at time scales that can range from minutes to decades. Moreover, evidence in humans and nonhuman animals suggests that NA also plays a role in such associative learning processes, potentially contributing to the biases that predict psychopathology (Figure 2).

## 3. Associative Learning in Humans and Nonhuman Animals

Associative learning is used as an umbrella term to refer to different types of learning that are characterized by the development of conscious or unconscious associations between a certain cue or action and the occurrence of a specific stimulus. For example, in an aversive *classical conditioning* paradigm, an animal learns to associate an initially neutral stimulus (CS+) with an aversive stimulus or event (US) that elicits an innate response [60]. After learning, the presentation of the CS+ alone leads to the aversive response. In *operant conditioning* or *reinforcement learning*, an animal learns that performing a certain action (e.g., pressing a lever) is followed by a specific outcome (e.g., delivery of food reward). Similar paradigms have been developed to study associative learning in humans. In the following paragraphs, we will review research on aversive and appetitive conditioning in both human and nonhuman animals, focusing on the role of NA and its relation to psychopathology.

### 3.1. Aversive Conditioning

The study of aversive conditioning in nonhuman animals has a long history of employing mild electric shocks as US and tones or lights as typical CS+ stimuli. Robust conditioning can be achieved after only a few continuous pairings of the CS with the US. Aversive conditioning in humans can employ a wide range of possible CS and US [61], and the extent of associative learning can be assessed by skin conductance response (SCR), eye blink reflex, and subjective stimulus ratings [1]. Aversive associations can also be learned quickly through instrumental or operant conditioning, in which subjects learn that a certain action will be followed by an aversive event. Studies of aversive conditioning have become essential for understanding the emergence of fear and fear-related disorders [62] and are important in identifying individual differences underlying susceptibility to anxiety disorders [63].

### 3.1.1. Neurocircuitry Underlying Aversive Conditioning

The brain circuitry underlying aversive conditioning is also quite well mapped. Research in nonhuman animals as well as lesion and neuroimaging studies in humans has identified the amygdala, the hippocampus, and the ventromedial prefrontal cortex as key nodes in brain systems underlying aversive conditioning. The amygdala plays a role in integration of information about CS and US and controlling fear responses via projections to autonomic and endocrine control systems in the brainstem [62]. Lesions of the amygdala are associated with impairments in both cue and context conditioning. In contrast, targeted lesions of the hippocampus lead to impaired context conditioning but not simple cue conditioning [64], indicating a dissociation between the roles of these two structures. The VMPFC is not only involved in extinction of learned fear by suppression of amygdala activity through interneurons [65, 66] but has also been shown to modulate fear-related activity in the amygdala and play an essential role in modulating fear expression [67]. Critically, this set of brain regions receives dense noradrenergic projections from the LC [68, 69].

### 3.1.2. The Role of NA in Aversive Conditioning—Relation to Psychopathology

Alterations in this circuit mediated by the LC-NA system are thought to underlie maladaptive patterns of fear learning expressed as fear and anxiety disorders such as PTSD [70, 71]. Fear learning is of course highly adaptive and critical for animals' well-being and survival. In situations of potential or actual threat or danger, rapid fear and defense mechanisms—including the release of NA and stress hormones—are activated [72, 73]. However, fear and stress responses are adaptive only when the timing and level of their activation are appropriate to the situation. A dysregulation of fear response or defensive behaviour can develop into a fear or anxiety disorder [74]. For example, posttraumatic stress disorder (PTSD) is an anxiety disorder characterized in part by attentional biases to mild stressors or cues related to the traumatic event that gave rise to the disorder as well as intrusive memories of the traumatic event [12, 75]. Pavlovian fear conditioning has been widely used as an animal model for PTSD contributing to the current understanding of the disorder [13]. Animal models of fear conditioning and human studies with PTSD patients and healthy controls provide evidence for a critical role of NA in this example of disordered fear learning. For example, patients with PTSD show greater baseline cerebral spinal fluid (CSF) NA concentrations [76] as well as elevated CSF NA levels after exposure to trauma-related material [77]. Much research on NA and PTSD has focused primarily on symptoms of the disorder or the fear response. For example, human studies found that the administration of the alpha2-adrenergic antagonist yohimbine (resulting in enhanced NA release) led to increased anxiety in patients with PTSD but not in control subjects [78]. Similarly, human PTSD symptoms have been alleviated by blocking NA activity: “beta blockers,” which reduce activity of beta-adrenergic receptors, have been demonstrated to be effective to reduce symptoms of anxiety in PTSD [79]. Convergent findings have demonstrated that pharmacological activation of inhibitory autoreceptors or blockade of postsynaptic alpha-1 adrenoceptors normalized exaggerated startle responses to contextual reminders of stress in a rodent model of PTSD [80]. Similarly, more recent human research has demonstrated that application of alpha1-adrenergic antagonists has been further shown to reduce psychological distress to trauma-related cues [81], and noradrenergic antidepressants have been demonstrated to be more successful than serotonergic antidepressants especially in patients with comorbid alcohol dependence [82]. Moreover, carriers of the *ADRA2b* deletion variant showed greater susceptibility to intrusive traumatic memory than noncarriers, suggesting a role for these receptors in the intrusive memory component of PTSD [45].

While one long-prevalent idea has been that PTSD results from disturbances in memory consolidation [83]—a process that has been shown to be highly modulated by NA [84]—recent intensification of interest in memory reconsolidation [85] has sparked new research in the field of PTSD and NA. Memory reconsolidation describes the process by which reactivation of a memory makes it modifiable. The potential to harness reconsolidation processes to manipulate traumatic memory is promising for the treatment of PTSD given its common resistance to extinction. Critically, it has been shown that beta-adrenergic stimulation of the amygdala after retrieval can enhance memory reconsolidation of fear memories, which makes them resistant to extinction, suggesting that noradrenergic activity during retrieval is likely to contribute to the formation of fear memories [86]. In turn, blockage of reconsolidation by alpha2-adrenergic agonist clonidine (resulting in reduced NA levels) has been shown to disrupt fear-related memories [87]. Thus, there is substantial convergent evidence linking PTSD, as an example of a disorder thought to be the result of disrupted fear learning, to altered noradrenergic transmission in fear learning and possible memory modulation. We speculate that NA-modulated alterations in fear learning observed in patients with PTSD may give rise to robust attentional biases for trauma-related cues observed in patients [12], demonstrating that specific affectively biased attentional sets develop as a result of individual differences in associative learning. Future research should test this hypothesis directly. While assessing NA activity in vivo in humans has been highly challenging to date (the LC is too small and variable between individuals to be reliably located with MRI [88]), pupil dilation is being found to be a relatively reliable index of LC activity [89–91], and imaging of neuromelanin has been recently employed as a measure of individual differences in LC structure [92, 93].

### 3.2. Appetitive Conditioning

Appetitive conditioning is an associative learning process by which initially neutral stimuli or events become associated with a reward and hence gain motivational salience (Figure 2). In appetitive classical conditioning, the presentation of a cue (CS+) becomes passively associated with a reward (US). Reward learning is more often studied in the form of appetitive operant conditioning or reinforcement learning. Here, a reward is obtained after the animal performs a certain action, which is hence reinforced [94]. Operant conditioning is thought to be driven by two distinct processes. Investigating the temporal dynamics of these processes is critical for the understanding of psychopathology related to reinforcement learning such as the development of addictive behaviours [95]. Early in the learning process, animal behaviour is predominantly goal directed; the animal performs the action leading to a reward (e.g., drug taking), the action-outcome association is developed [96]. Later behaviour becomes much more habitual or even compulsive, that is, that no longer the reinforcing property of the reward (e.g., the drug) leads to action completion but the action is performed irrespective of the actual outcome and even despite negative consequences [97]. Critically, this shift in behaviour has been shown to be promoted by glucocorticoid and NA release as part of the stress response [98, 99] (Figure 2). Neuroimaging data suggest that NA and glucocorticoid action disrupt the neural basis for goal-directed behaviour [100]. The authors report that under influence of these stress hormones, the OFC became insensitive to changes in outcome value while brain regions related to habit behaviors (e.g., dorsal striatum) were unaffected allowing those behaviours to take over under acute stress.

### 3.2.1. Neurocircuits Underlying Appetitive Conditioning

Converging evidence from human and nonhuman studies suggests that the amygdala plays a key role in appetitive conditioning. The amygdala has been shown to be critical for outcome evaluation and cost estimation [101, 102] as well as for the development of CS-US associations and attentional modulation in reward processing [103–105]. Due to its rich connections with the OFC and striatum, the BLA is also important for integration and relay of information allowing for flexible, goal-directed behaviour [95, 101, 103]. The OFC in turn receives information from the amygdala and is central for reward evaluation and outcome expectancies [106]. Besides the OFC, the anterior cingulate cortex (ACC) has been shown to be an essential node of circuitry required for normal contingency learning [107] as well as for the discrimination of multiple conditioned stimuli [108]. The striatum has been suggested to play a general role in the processing of stimulus salience [109] and is also of major importance for the formation of habits [110] and hence for psychopathology associated with appetitive learning. The central role of dopaminergic action in the ventral striatum with projections to the prefrontal cortex and amygdala is well established and has been extensively reviewed elsewhere [111–113]. However, this set of brain regions also receives dense noradrenergic projections from the LC [68, 69] and displays a high density of alpha2-adrenergic receptors [114]. As mentioned above, due to its small size and considerable variability in location, LC activation has been challenging to measure with common neuroimaging methods such as fMRI [115]. However, from animal research, it has long been known that the LC displays conditioned responses after only a few learning instances for both aversive as well as appetitive reinforcers [116] as further discussed in the next section.

### 3.2.2. The Role of NA in Appetitive Learning—Relation to Psychopathology

Increasing evidence suggests that the LC-NA system not only is important for aversive conditioning but also plays a role in reward processing related to addiction. Decades of research have established that dopamine (DA) is essential for the reinforcing effects of various rewards such as drugs [117–119]. A selective role of DA in reward learning has been shown to be that of a mediator of incentive salience that is the motivational properties that a stimulus develops through conditioning [112, 118]. In other words, DA has been shown to be essential for the “wanting” of a reward, but not for the associated pleasure, or “liking,” or for the associative learning process. Furthermore, DA has been shown to be a key for the coding of reward prediction errors, operationalized as the difference between anticipated and actual reward [120]. In contrast, the contribution of NA has been relatively neglected [121] despite its abundance throughout the brain and its central role in arousal, attention as well as cognitive flexibility and adaptation [27, 35]. However, recent investigations have linked activation of the noradrenergic system to motivation. NA has been shown to be important for morphine-associated conditioned place preference (CPP) [122] as well as its rewarding effects [123]: decreasing noradrenergic activity (by stimulating alpha2-adrenergic autoreceptors) inhibits the development of CPP, while enhancing NA availability (by receptor inhibition) facilitates conditioning for actual reward learning processes. Previous research has further demonstrated that if NA transmission in the mPFC is blocked, DA release in the nucleus accumbens in response to morphine or amphetamine is abolished, suggesting that prefrontal NA has a central role in the rewarding effects of some drugs [124, 125]. The authors speculate that this effect can be explained by blocking NA effects on the striatum via three distinct routes: NA activates (1) excitatory projections to the ventral tegmental area, (2) glutamatergic projections to the nucleus accumbens, and (3) GABAergic neurons controlling DA neurons through double inhibition. Thus, in this instance, NA may work as a control instance-mediating reward-associated dopaminergic activity. Future research has to be conducted to provide evidence for this hypothesized role. A series of single-cell recording studies conducted in monkeys by Bouret and Richmond further supports the involvement of the LC-NA system in reward learning. Single-cell recordings from LC neurons during a task with both Pavlovian and operant components revealed that LC neurons are activated during conditioned responses and their response is modulated by goal-directed processes [126]. Directly comparing activity of noradrenergic LC and dopaminergic substantia nigra pars compacta neurons suggests that these neurotransmitters play slightly different roles, with DA responding to rewarded actions—possibly related to value—while NA neurons fire in response to unrewarded action, potentially suggesting it signals the cost associated with an action [127]. More recent research further suggests that the LC plays a role in reward processing by integrating motivationally relevant information such as cue information and reward size [128]. The authors extend their interpretation of the results to conclude that the LC is necessary to trigger actions requiring a high amount of energy because the incentive salience is low. This idea is supported by their findings showing that noradrenergic neurons increase their firing rate with increased effort in an effort-based decision-making task [91]. That is, LC activation is necessary to produce behavioural energy in such a task after a cost-benefit analysis, while dopaminergic activity codes information about the costs and benefits involved. Empirical evidence further suggests that the LC might be related to environmental uncertainty. In an fMRI study, phasic pupil diameter as a proxy for LC activity correlated with uncertainty during learning in a predictive-inference task [129]. In contrast, another study revealed a negative response to unexpected uncertainty in the LC while human participants performed a decision-making task [130]. The authors speculated that theses conflicting results could be explained by the characteristics of phasic LC mode. Phasic firing has been associated with enhanced task engagement [35] and involves both a decreased baseline firing rate as well as increased phasic responding to task-relevant stimuli [130]. Thus, while the results of the first study fall in line with the predicted association of phasic firing rate and task performance, the results of the second study suggest that the signal observed under conditions of high uncertainty reflect baseline activity [130]. As summarized in a recent theoretical paper, this empirical evidence supports the idea that the LC-NA system may work as an uncertainty signal-driving behaviour to adapt to environmental changes [131]. Extrapolating from these findings, we propose that the activation of the LC-NA in situations of uncertainty with respect to reward expectations facilitates attentional biases for reward-related cues (Figure 2). Such biases in turn allow for more efficient and eventually habitual tracking [59] of cue-outcome relations. Failures of reward evaluations may give rise to the excessive attentional biases for reward-related cues that have been found to characterize addiction [59].

Putative neuronal mechanisms underlying the role of LC-NA in attentional mechanisms related to reward have been further elucidated in a recent study suggesting a major role of the LC-NA system in modulating neural gain [34]. Under some circumstances, increased gain, which is associated with greater NA availability, narrows attention to those categories of stimulus that individuals are already predisposed to attend to and strengthens only the strongest neural connections. As a result, behaviour can become more rigid, flexibility can be impaired, and habitual behaviours are favored [34]. This model is in line with an existing theory relating the LC-NA system to neural gain [35] as well as with empirical evidence showing that pupil diameter as an index of LC activity predicts exploration versus exploitation between individuals as well as across trials [132]. The model has important implications for reward learning as it can explain the described shift from goal-directed to habitual behaviour. Such a shift observed upon simultaneous noradrenergic and glucocorticoid action [100, 133] and is prevented when noradrenergic activity is blocked [134]. That is, under conditions of high gain or high NA levels, behaviour shifts from flexible, goal-directed behaviour to more rigid, habitual control of behaviour. It is no longer the rewarding outcome driving ones' behaviour but simple stimulus-response mechanisms that have been established [133]. It also proposes neural mechanisms underlying the development on habitual or automatic attentional biases from reward learning [59]. Future studies employing convergent techniques to manipulate and measure NA activity in humans, such as pupil dilation [89], stress induction, pharmacological challenges, and genotyping, will be necessary to further investigate the role of NA in appetitive conditioning and its relevance for psychopathology.

A prevailing view in the addiction literature is to characterize addiction as a disorder of appetitive learning [97]: On the one hand, drugs act as reinforcers, such that the rewarding effect of the drug leads to enhanced drug taking. On the other hand, environmental stimuli that become associated with the drug effects can acquire incentive salience through Pavlovian conditioning [95]. An important component of addiction is an imbalance of goal-directed and habitual behaviours. In the beginning, drug taking or substance use is a goal-directed process guided by the reinforcing properties or the “liking” of the drug. However, over time behaviour can shift towards the habitual. That is, “wanting” or craving for the substance develops irrespective of the rewarding outcome and often despite accompanying negative consequences—a process shown to be dependent on dopaminergic action [117]. Thus, instead of relying on action-outcome relations, addicts show a high degree of stimulus-response instrumental responding. Support for this idea can be found in both human and nonhuman animal research (for review, see [97]). These findings raise the question of what determines whether behaviour shifts from goal directed to habitual and what may make some people more prone to experience the shift. We propose that the LC-NA system contributes to this shift and that individual differences in NA availability may underlie differences in vulnerability to addictive habits (Figure 2). As described earlier, in some contexts, high NA levels have been associated with more rigid, less flexible behaviour [34]. Thus, either transient elevation of NA levels (e.g., by acute stress) or altered NA availability based on genotype (e.g., *ADRA2b* polymorphism) may explain greater predisposition to maladaptive habit formation observed in some individuals. In fact, both human and nonhuman studies have revealed that chronic or acute stress can bias behaviour towards the habitual [98, 135, 136] adding to the literature showing that acute stress—and resultant NA and corticosteroid action—elevates drug self-administration and promotes relapse [137, 138]. Pavlovian learning has also been shown to be a factor in drug addiction since environmental and drug-related cues can promote craving, drug taking, and relapse [97]. As described earlier, associative learning can largely modulate attentional biases—for example, to drug-related cues—which in turn guide or control our behaviour. Biases to those reward-related cues, which become habitual based on learned associations [59], can in turn inform instrumental behaviour through Pavlovian-instrumental transfer (PIT), in which an initially neutral cue that becomes associated with the drug may elicit instrumental or habit behaviour such as drug taking (Figure 2). Critically, PIT has likewise been demonstrated to be promoted by acute stress [139] and thus is likely influenced by NA-related processes. Yet, whereas empirical evidence points towards an involvement of the LC-NA system in normal reward learning, evidence for a role of the LC-NA system in addiction is sparse [140].

While addiction is characterized by attentional biases associated with increased approach motivation, the opposite picture is present in patients with major depressive disorder (MDD). Anhedonia—the inability to experience pleasure—is a cardinal symptom of depression [141, 142]. Importantly, anhedonia is characterized by reduced attentional biases to reward [143]. This again is thought to be due to altered patterns of associative learning observed in depression [144–146]. A number of studies have suggested that patients with depression display a deficit in approach motivation are less responsive to rewards and show reduced activation in reward circuitry (for review, see [147]). A recent study employed a computational meta-analysis to formalize the relation between anhedonia and reinforcement learning and to answer the question of whether MDD patients simply show reduced reward sensitivity or whether the ability to learn from a reward signal is impaired [148]. The results suggested that the actual learning rate—that is, the speed with which the action-outcome association is established—is not affected in patients with depression. However, patients show overall reduced effort and willingness when working for the same reward as controls, suggesting that their reward sensitivity is reduced. Besides its direct relevance for the psychopathology of anhedonia, these findings also suggest that reward-related learning has at least two distinct contributions: learning rate and reward sensitivity [148]. This distinction is critical for our understanding of how associative learning informs attentional biases. Consistent with the proposed link between attentional biases and associative learning processes, patients with anhedonia display altered reward learning as well as reduced attentional biases [149, 150]. This suggests that altered learning processes indeed give rise to differences in attentional prioritization related to psychopathology. In line with the above proposed role of NA in reward learning, there is additional evidence that acute stress, as a natural stimulator for NA and glucocorticoid release, affects reward sensitivity [151–153]. It is critical to point out that based on current research, noradrenergic processes are not easily distinguishable from the involvement of the dopaminergic and serotonergic system. The goal of this review is to propose the LC-NA system as an additional factor contributing to the pathological alterations observed.

In summary, a large body of literature suggests that NA-mediated alterations and individual differences in the appetitive associative learning system give rise to specific patterns of biased attention. Attentional biases can both be strengthened (e.g., addiction) and weakened (e.g., depression) through reward learning and can develop into deeply habitual patterns of orienting to the world that underlie the etiology and maintenance of psychopathology.

## 4. Conclusion

In summary, we have argued that NA plays an important role in the genesis and maintenance of biased attention patterns that are established via associative learning processes. Here, we first reviewed evidence for the emergence of attentional biases linked to psychopathology in development and the role of putative individual differences in NA availability in such biases. We next reviewed associative learning processes that can give rise to such biases, as well as evidence suggesting a role for NA in specific patterns of fear learning linked to PTSD and appetitive learning linked to both addiction and depression. Based on convergent evidence, we propose that attentional biases play a key role in creating and maintaining prioritization of relevant cues as well as the transfer of reward learning to habitual behaviours associated with addiction. We hypothesize that after attentional biases for reward-related cues are formed through associative learning processes, they are themselves used to inform and prompt behaviours. More specifically, they may facilitate the formation of habitual behaviours by redirecting attention from the outcome to the cue. This is a possible mechanism that could explain why habitual behaviours are performed even if the outcome changes towards the negative. In addition, such biases themselves form deeply habitual patterns of orienting to the world, which can play an important role in etiology and maintenance of psychopathology.

## 5. Future Directions

A number of outstanding questions remain. First and foremost, little is known about the role of NA in appetitive learning in humans. While previous research in humans demonstrated a role of stress in habit formation and Pavlovian-instrumental transfer, it remains to be investigated whether initial reward learning is affected by NA availability. Future research can examine this by manipulating NA availability, for example, through acute stress induction or by using the *ADRA2b* genotype as a source of naturally occurring differences in NA availability. It will be important to delineate how both operant and Pavlovian conditioning are affected by these manipulations and whether it is actual learning rate or reward sensitivity that is affected. Future research should aim to disentangle these two components of reward learning. If stress is used as a means to activate the LC-NA system, the intensity and type of stressor need to be considered [154]. Effects of stress are most likely to be observed when the stressor acts on those brain regions that are involved in task completion [154, 155]. The effects of varying stress levels are best represented in the well-established inverted U curve of arousal, which indicates that performance is best at intermediate stress or arousal levels while both low and high stress levels have a relative negative impact [156, 157]. Thus, the level of arousal, as well as the source of stress, will play a crucial role in both the general effects of NA on learning as well as their translation into attentional biases.

Moreover, the proposed link between associative learning and attentional biases needs to be tested directly in humans. That is, once the role of NA in associative learning is fully established, one should examine whether newly learned associations result in attentional biases for cue- or outcome-related stimuli.

In addition, the directionality of the proposed link needs to be investigated further. While converging evidence suggests that associative learning processes form attentional biases, attentional biases are likely to influence later instances of emotional learning. It is unclear whether activity of the LC-NA would further reinforce existing biases by influencing subsequent learning processes or whether one of main roles of this neurotransmitter system is to facilitate learning processes that give rise to attentional biases. It is likely that the process can be mediated at both ends; however, this problem needs to be investigated in more detail.
